# Physical Proximity May Promote Lateral Acquisition of Bacterial Symbionts in Vesicomyid Clams

**DOI:** 10.1371/journal.pone.0064830

**Published:** 2013-07-08

**Authors:** Carole Decker, Karine Olu, Sophie Arnaud-Haond, Sébastien Duperron

**Affiliations:** 1 IFREMER Centre de Brest, Laboratoire Environnement Profond, REM-EEP, Plouzané, France; 2 UMR 7138 (UPMC CNRS IRD MNHN), Systématique Adaptation Evolution – Adaptation aux Milieux Extrêmes, Paris, France; 3 Université Pierre et Marie Curie, Paris, France; Université Paris Sud, France

## Abstract

Vesicomyid clams harbor intracellular sulfur-oxidizing bacteria that are predominantly maternally inherited and co-speciate with their hosts. Genome recombination and the occurrence of non-parental strains were recently demonstrated in symbionts. However, mechanisms favoring such events remain to be identified. In this study, we investigated symbionts in two phylogenetically distant vesicomyid species, *Christineconcha regab* and *Laubiericoncha chuni*, which sometimes co-occur at a cold-seep site in the Gulf of Guinea. We showed that each of the two species harbored a single dominant bacterial symbiont strain. However, for both vesicomyid species, the symbiont from the other species was occasionally detected in the gills using fluorescence *in situ* hybridization and gene sequences analyses based on six symbiont marker genes. Symbiont strains co-occurred within a single host only at sites where both host species were found; whereas one single symbiont strain was detected in *C. regab* specimens from a site where no *L. chuni* individuals had been observed. These results suggest that physical proximity favored the acquisition of non-parental symbiont strains in Vesicomyidae. Over evolutionary time, this could potentially lead to genetic exchanges among symbiont species and eventually symbiont displacement. Symbiont densities estimated using 3D fluorescence *in situ* hybridization varied among host species and sites, suggesting flexibility in the association despite the fact that a similar type of metabolism is expected in all symbionts.

## Introduction

Vesicomyid bivalves are one of the most abundant groups of chemosynthetic fauna inhabiting deep-sea reducing ecosystems, including hydrothermal vents, whale falls and cold seeps [1]–[3]. More than 100 living species have been described worldwide, mainly at bathyal and abyssal depths [4], [5]. Vesicomyid bivalves live in symbiosis with sulfur-oxidizing bacteria located in their gills [6], [7]. These symbionts produce organic compounds used by the bivalve hosts for their nutrition, and in turn, the bivalve hosts supply the symbionts with oxygen, carbon dioxide and reduced sulfur. Oxygen and carbon dioxide are directly available in the ambient seawater and are transported, through the inhalant siphon, to gills and bacteria therein. Sulfide from the sediment is absorbed in the host through the foot into the hemolymph and transported to the gills [8].

Two species, *Christineconcha regab* Cosel and Olu, 2009 [4], [9] and *Laubiericoncha chuni* Thiele and Jaeckel, 1931 [10], occur at the Regab cold-seep site, a giant pockmark located at 3150 m depth in the Gulf of Guinea [4], [11], [12]. Co-occurrence of two chemosymbiotic bivalve species at a single seep site raises questions regarding potential interspecific competition, symbiont specificity, and niche partitioning. Species may display slightly different optima in terms of oxygen or fluid utilization, or slightly different physiology allowing them to co-exist with limited competition. However, considering the importance of symbionts in vesicomyid metabolism, partitioning may also be achieved through the association with slightly different symbiont strains. For example, differences in sulfide physiology have been demonstrated in two vesicomyids (*Calyptogena pacifica* and *Phreagena kilmeri*) co-occurring at cold-seep sites in Monterey Bay, USA [13].

Symbionts are thought to be vertically transmitted from parents to offspring in vesicomyid clams [14], [15], and each host species is typically associated with a single particular bacterial strain. However recent studies have demonstrated occasional leaks in this vertical transmission, leading to lateral acquisition (*e.g.* horizontal transfer between unrelated hosts or environmental acquisition) of non-parental symbiont types [16]. In addition, genomic analyses suggest that genome recombination can occur among distinct symbiont lineages [17]. Physical proximity of distinct species co-occurring in the same site may favor incidental exchange of symbiont strains or genes, although this remains to be demonstrated.

Approaches involving multiple bacterial genes are required to test for the occurrence of unexpected symbionts (*i.e.* a bacterial species different from the main symbiont strain associated with a given host species), or even the co-occurrence of multiple symbiont strains. Multiple markers are necessary to distinguish actual co-occurrence of multiple symbiont strains in a single individual or host from simple gene recombination. The presence of distinct symbiont strains and their distribution within host tissues can be further studied by fluorescence *in situ* hybridization (FISH) using 16S rRNA strains-specific probes.

In this study, we tested whether host species harbor a specific strain or multiple symbiont strains in two phylogenetically distant [18] vesicomyid host species, *Christineconcha regab* and *Laubiericoncha chuni*, collected from a West African pockmark. We screened six bacterial loci to look for footprints of strain occurrence and genetic recombination among symbiont strains at sites where both species co-occur. A barcoding approach based on metazoan cytochrome c oxidase subunit I (COI) was used to confirm morphological identification of vesicomyid specimens. Associated symbionts were identified using two genes (encoding 16S rRNA and 23S rRNA) and localized in gill tissue using FISH with strain-specific 16S rRNA-targeting probes. Multiple locus sequence typing (MLST) and phylogeny was employed on six symbiont genes to screen for evidence of potential recombination. Finally, from a more ecological point of view, we investigated whether sites or host species influence the density of bacterial symbionts measured using 3D FISH as the percentage of bacteriocyte volume occupied by symbiont bacteria [19]. This study thus aims to help elucidate the potential role of symbionts in the co-occurrence of closely related species.

## Materials and Methods

No specific permits were required for the described field studies.

### 1. Specimen collection

The giant pockmark Regab (800 m wide and 15 m deep) was discovered in 2001 at 3160 m depth along the Congo-Angola margin [11] and re-explored with the ROV *Quest 4000* (MARUM) during the 2008 Guineco cruise, leg M76/3b in West Africa, aboard the R/V *Meteor* (chief scientist: A. Boetius). Dense assemblages of symbiont-bearing species had been previously documented, including the Bathymodiolinae species *Bathymodiolus* aff. *boomerang* [20], the Vesicomyidae species *Christineconcha regab* and *Laubiericoncha chuni*, and the Siboglinidae polychaete species *Escarpia southwardae* Andersen et al., 2004. Mytilids and siboglinids dominate in the center of the pockmark, and the periphery is dominated by vesicomyid beds [11], [12]. No major changes in this general distribution pattern were observed during ROV surveys in 2008 (C. Decker, K. Olu, pers. obs.), or in 2011 [21]

**Figure 1:**
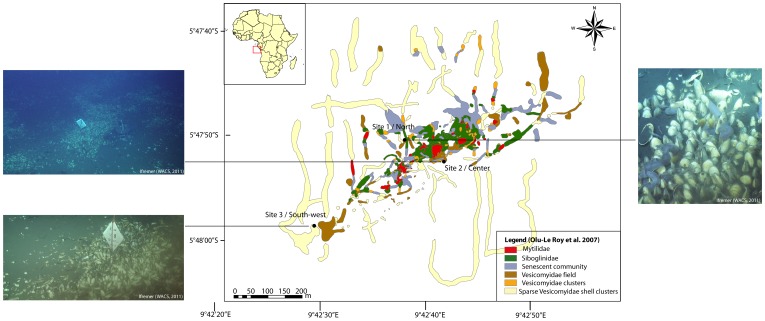
Location of the three study sites on the Regab pockmark: Site 1 at the north of the pockmark (Guineco Marker 3), Site 2 at the center of the pockmark (Guineco Marker 7) and Site 3 in the southwestern part of the pockmark (Guineco Marker 10). Images ROV *Victor 6000*, WACS cruise – Ifremer. Reprinted from Ifremer WACS cruise under a CC BY license, with permission from Ifremer, original copyright 2011.

Three sites were studied along a NE-SW axis: (1) a site in the north (Site 1) (2) a site at the center of the pockmark (Site 2) and (3) a southwestern site (Site 3) (Table 1, Figure 1). At Sites 1 and 2, vesicomyid clam beds were patchily distributed compared to Site 3 where a large, contiguous vesicomyid bed was observed.

**Table 1:** Study sites at the Regab pockmark and number of individuals sampled (Cr: *Christineconcha regab* and Lc: *Laubiericoncha chuni*).

Site (Marker)	Latitude	Longitude	Depth (m)	Dive Number	PANGAEA ID	No. of individuals sampled
1-North (M3)	S 5°47.838	E 9°42.6284	3167	209	M76_3b_312_NET1	5 Cr
1-North (M3)	S 5°47.838	E 9°42.6284	3167	211	M76_3b_ 323_NET1M76_3b_323_PUC12	10 Cr
2- Center (M7)	S 5°47.8674	E 9°42.6881	3171	217	M76_3b_344_NET2	4 Cr
3-South-West (M10)	S 5°47.9761	E 9°42.4825	3170	225	M76_3b_379_NET2	5 Cr+3 Lc

*C. regab* was sampled from all three sites, whereas *L. chuni* was rare and only observed and sampled in Site 3, along with *C. regab*. Both species were sampled using a ROV-manipulated net (ROV *Quest 4000*, MARUM).

**Figure 2:**
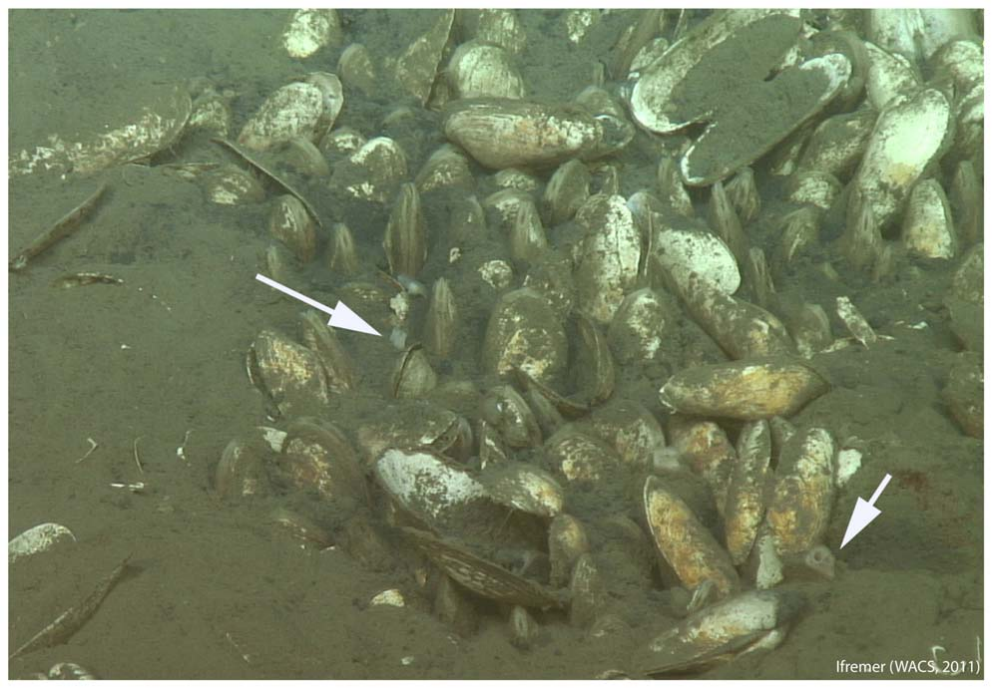
A vesicomyid bivalve bed in Site 3 (see Figure 1), with *C. regab* and *L. chuni.* Arrows show *L. chuni* siphons. Images ROV *Victor 6000*, WACS cruise – Ifremer. Reprinted from Ifremer WACS cruise under a CC BY license, with permission from Ifremer, original copyright 2011.

*C. regab* was visible at the surface of the sediment (Figure 2) and was characterized by short siphons, a single demibranch and clear hemolymph [9]. In contrast, *L. chuni* occurred deeper in the sediment, with only its long siphons visible on the surface [9] (Figure 2). This species has two demibranchs and red hemolymph.

The sample consisted of 15 *C. regab* specimens from Site 1, 4 *C. regab* from Site 2, and 7 specimens (4 *C. regab* and 3 *L. chuni*) from Site 3 (Table 1). All specimens were morphologically identified and dissected onboard. A part of the adductor muscle and the gill were frozen for DNA analysis, and the most anterior part of the gill was stored for FISH as described in Duperron *et al.* (2005) [22].

### 2. Molecular characterization of hosts and bacteria

Total DNA from the adductor muscle was isolated using the CTAB extraction method [23]. DNA was extracted from gills using the method described by Zhou et al (1996). Universal primers [24] were used to amplify a 710 bp fragment of the metazoan mitochondrial COI gene (Table S1).

The bacterial 16S rRNA-encoding gene, as well as six additional genes located in various regions of the symbiont genome, were amplified (23S rRNA, adenosine-5-phosophosulphate reductase protein (*aprA*), dissimilatory sulfite reductase beta subunit (*dsrB*), sulfur oxidation protein (*soxA*), bacterial cytochrome c oxidase cbb3-type subunit I (*ccb3*), bacterial *coxI* gene, see Stewart et al. (2009) [17]). PCR products were purified (QiaQuick Kit ™, Qiagen), cloned using a TA™ Cloning Kit (Invitrogen, CA), and sequenced in two to three randomly chosen specimens of each species at each sampling site. PCR products from all other specimens (17 specimens) were sequenced directly and did not show any ambiguous positions (*e.g.* double peaks). All primers and PCR programs used are summarized in Table S1. All amplified loci were sequenced in both directions by GATC Biotech (Germany). GenBank accession numbers are listed in Table S2.

### 3. Sequence analyses

Sequences were compared with sequences available in GenBank using BLAST [25]. Phylogenetic trees were constructed for each gene including the best BLAST hits and reference sequences. Because GenBank contains data obtained prior to the new taxonomic revision of Vesicomyidae, we renamed unidentified or misidentified specimens of sequences retrieved from GenBank according to their identification in the most recent taxonomic revisions by expert taxonomists [5], [9], [26], [27]. Consequently, some species belong to undetermined genera (*e.g.* ‘Undetermined genus’ *cordata* formerly *Vesicomya cordata*).

Alignments of nucleotide sequences were conducted using Geneious Pro 4.6 [28], and checked by eye to maximize positional homology. The Akaikes information criterion (AIC) [29] implemented in Modeltest v. 3.7 [30] was used to determine the evolutionary model that best fitted the data set. PhyML v. 2.4.4 [31] was used to estimate the maximum-likelihood (ML) tree using the selected model [32], and to test the robustness of the inferred clades using non-parametric bootstrap proportions (BPs, 1000 pseudo-replicates). Host *coxI* phylogeny was rooted with *Venus antiqua* and bacterial 16S rRNA and *aprA* phylogenies were rooted with sub-family Bathymodiolinae and family Thyasiridae symbionts respectively, because of their close relationships with vesicomyids.

### 4. Identification of symbionts using FISH

Gill tissue was dehydrated in an ethanol series, embedded in polyethylene glycerol disterate∶1-hexadecanol (9∶1) wax, and sectioned (10 µm) as described elsewhere [19]. Hybridizations were performed at 46°C for 3 h; sections were immersed in a buffer containing 100 ng of each probe in hybridization mix (0.9 M NaCl, 0.02 M Tris-HCl, 0.01% SDS, 30% formamide). Slides were then washed (0.1 M NaCl, 0.02 M Tris-HCl, 0.01% SDS, 5 mM EDTA) at 48°C for 20 min.

After identification of phylotypes based on 16S rRNA gene sequences, phylotype-specific probes were designed each displaying 100% match with their target strain, and a 22% difference with the non-target strain. They were first tested on specimens harboring only one 16S rRNA symbiont phylotype according to cloning and sequencing results. Probes were shown to be discriminant at 30% formamide (Table 2), in which they hybridized with most of the targeted bacteria present in gill cross-sections, and produced no signal in the non-target strain. Four to six gill cross-sections from nine different *C. regab* specimens (three per sampling site) and three *L. chuni* were dual-hybridized using the two probes with distinct fluorochromes (Cy-3 and Cy-5). Probe Eub338 was used to counter-stain some sections. Image stacks, consisting of a series of consecutive images taken every 0.48 µm over the thickness of the sections were obtained for each fluorochrome using an ApotomeAxio Imager Z2 with a COLIBRI system (Zeiss, Germany).

**Table 2:** FISH probes used in this study, percentage of formamide in hybridization buffer, and target groups.

Probe	Sequence (5′ – 3′)	% Formamide	Target	Reference
EUB338	GCTGCCTCCCGTAGGAGT	30	Most eubacteria	Amann *et al.* 1990 [59]
GAM42	GCCTTCCCACATCGTTT	30	Gammaproteobacteria	Manz *et al.*1992 [60]
Creg821	GTACCCCCCCCAACGACT	30	Thiotrophic symbionts of *Christineconcha regab*	This study
Lchu821	GTAAATCCCCCCAACGGCT	30	Thiotrophic symbionts of *Laubiericoncha chuni*	This study

### 5. Estimation of symbiont densities by 3D-FISH

For each specimen (three specimens per species per site), symbiont densities were estimated using 3D FISH as the percentage of gill volume occupied by the bacteria strain. On 48 gill sections, a total of 720 square subsections of 100×100 µm and 10 µm thickness (12 specimens×4 sections per specimen×15 squares per section) were sampled randomly and the fraction of total volume occupied by bacteria was computed using the SYMBIONTJ application [19] (http://www.snv.jussieu.fr/%7Ewboudier/softs/symbiontj.html) implemented in ImageJ [33].

Percentages of volume occupied by bacteria were analyzed using R software [34] after an arcsine transformation, and compared between species and sites with a non-parametric test (Kruskal-Wallis test if n2 and Wilcoxon-Mann Whitney test if n = 2).

## Results

### 1. Host COI sequences

COI was sequenced for all sampled specimens, and confirmed the morphology-based identification. The 23 specimens identified onboard as *C. regab* yielded highly similar sequences (four haplotypes with 0 to 0.6% difference over 631 bases). A single COI haplotype was detected in the three *L. chuni* specimens. Sequences from both species differed by a mean of 11.5%. *C. regab* sequences displayed 92% similarity with its closest relative *Isorropodon perplexum*, whereas the unique *L. chuni* sequence was very similar to that of *Archivesica* cf. *angulata* (formerly *V.* sp. of Sanriku) from Japan (2% divergence). *C. regab* and *L. chuni* are thus sympatric species that belong to distinct clades in the larger vesicomyid phylogeny (Figure S1) [18].

### 2. Bacterial 16S rRNA

**Figure 3:**
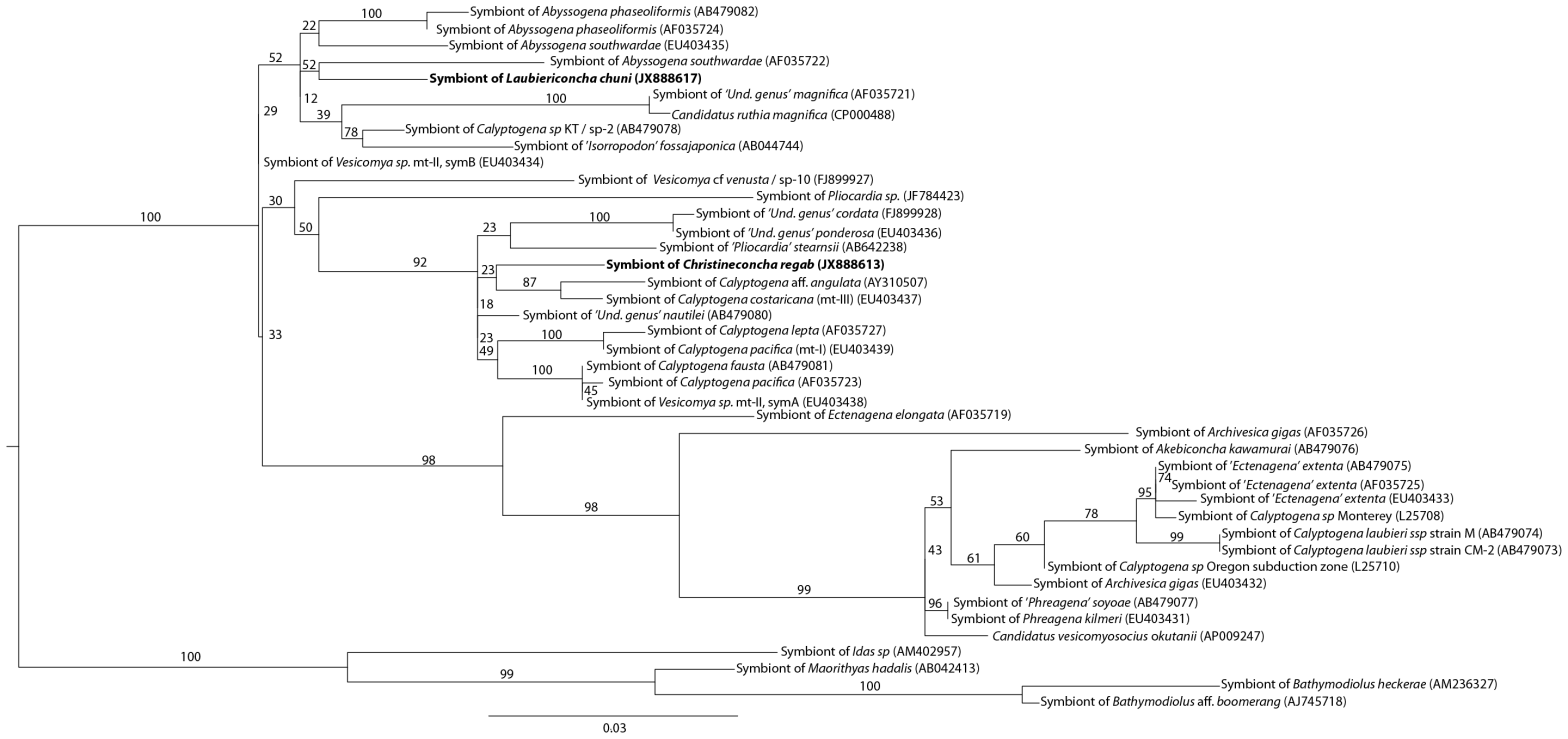
16S rRNA maximum-likelihood tree. The evolutionary model tested was GTR+I+G (proportion of invariable sites = 0.66, number of substitution rates = 6, gamma distribution parameter = 0.64). Bootstrap values for 1000 replicates are given in percent above branches and clades. Scale bars are expressed as number of substitutions per base pair. ‘Und. genus’) indicates a temporary genus name. Sp-1 to 9 correspond to species numbers given in Audzijonyte *et al.* 2012.

A total of 137 clone and 17 direct sequences were analyzed for 16S rRNA (Table 3). With the exception of two specimens (217-V3 and 225-V1), a single 16S rRNA phylotype was recovered for each species (Table 3). Sequences identified in *C. regab* and *L. chuni* differed by 1.6%, and both displayed above 96% similarity with sequences from several other vesicomyid symbionts. Both sequences clustered within the clade that includes all vesicomyid-associated sulfur-oxidizing gammaproteobacterial symbionts (Figure 3). However, the two symbiont strains at Regab belonged to distinct clusters. The symbiont of *C. regab* clustered with symbionts from Pacific seep vesicomyids and ‘Undetermined genus’ *cordata* and ‘Undetermined genus’ *ponderosa* from the West Atlantic (98.9% similarity with the symbiont of ‘Undetermined genus’ *nautilei*). In contrast, the symbiont of *L. chuni* showed the highest similarity to symbionts from *Vesicomya* sp. mtII (symB, East Pacific) and ‘*Calyptogena*’ sp. KT from the Japan Trench (2.5% divergence) (Figure 3). In specimens 217-V3 (*C. regab*) and 225-V1 (*L. chuni*), both 16S rRNA phylotypes co-occurred in clone libraries, with over 90% of clones corresponding to the expected, species-typical symbiont strain (Table 3).

**Table 3:** Results of direct sequencing (red for sequences from the *C. regab* symbiont and blue for the *L. chuni* symbiont), and cloning*.

Site	Specimen	Species	Marker gene						
			16S (98.4%)	23S (98.6%)	*aprA* (95.1%)	*dsrB* (92.8%)	*soxA*(90.6%)	*coxI*(97.4%)	*cbb3* (94%)
1- North	209 V2 to V5	*C. regab* (n = 13)	x	x	x	x	x	x	x
	211 Net5 V1 to V3 and V5								
	211 PC12 V1 to V5								
	209 V1*	*C. regab*	100 (4)	100 (36)	100 (12)	100 (4)	100 (10)	100 (7)	100 (5)
	211 Net5 V4*	*C. regab*	100 (6)	100 (44)	100 (10)	100 (23)	100 (11)	100 (4)	100 (1)
2- Center	217 V4 and V5	*C. regab* (n = 2)	x	x	x	x	x	x	x
	217 V2*	*C. regab*	100 (4)	100 (35)	100 (12)	100 (9)	100 (10)	88 (7); **12 (1)**	100 (4)
	217 V3*	*C. regab*	91 (32); **9 (3)**	100 (53)	86 (47); **14 (8)**	100 (21)	100 (22)	ND	100 (1)
3- Southwest	225 V2 and V4	*C. regab* (n = 2)	x	x	x	X	x	x	x
	225 V3*	*C. regab*	100 (6)	100 (33)	100 (12)	100 (8)	100 (8)	**100 (6)**	100 (5)
	225 V5*	*C. regab*	100 (38)	100 (64)	70 (45); **30 (17)**	100 (23)	40 (2); **60 (3)**	ND	100 (3)
	225 V1*	*L. chuni*	**95 (19)**; 5 (1)	**100 (58)**	**64 (33)**; 36 (19)	**100 (19)**	**100 (8)**	**100 (7)**	**100 (15)**
	225 V6*	*L. chuni*	**100 (9)**	**100 (34)**	**100 (13)**	**100 (17)**	**100 (11)**	**86 (6)**; 14 (1)	**100 (13)**
	225 V7*	*L. chuni*	**100 (15)**	**100 (46)**	**100 (12)**	**100 (9)**	**100 (8)**	**100 (17)**	**100 (11)**

Percentage of divergence between the two symbiont sequences is given in parentheses in the header after each locus name. Given are details on the percentage (number) of sequences from *C. regab* and *L. chuni* symbiont (**bold**), and the number of clones sequenced in parentheses. ‘x’ indicates sequences obtained without cloning.

### 3. Other bacterial genes

A total of 986 clones and 102 direct sequences were analyzed for other genes on symbionts from two to three specimens of each vesicomyid species at each site, namely bacterial 23S rRNA, *aprA*, *dsrB*, *soxA*, *ccb3* and *coxI*. Co-occurrence of symbiont phylotypes in a single host was detected in only one specimen of each species based on 16S rRNA sequences (Table 3). The use of multiple markers revealed the presence of two strains in other specimens that exhibited a single, expected 16S rRNA phylotype (Table 3). Each specimen only harbored a single unique sequence for 23S rRNA, *dsrB* and *cbb3*. For *aprA* three specimens, 217-V3 and 225-V5 for *C. regab*, and 225-V1 for *L. chuni*, harbored both observed sequences. For *soxA*, only one *C. regab* specimen (225-V5) harbored both sequences. For *coxI*, specimens 217-V2, 225-V3 (*C. regab*) and 225-V6 (*L. chuni*) harbored both sequences. In all, specimens for which two distinct sequences co-occurred for one or more gene, the sequence expected to be found in the corresponding host species was dominant in the clone library, the only exception being the gene *dsrB* in specimen 225-V5 (Table 3).

**Figure 4:**
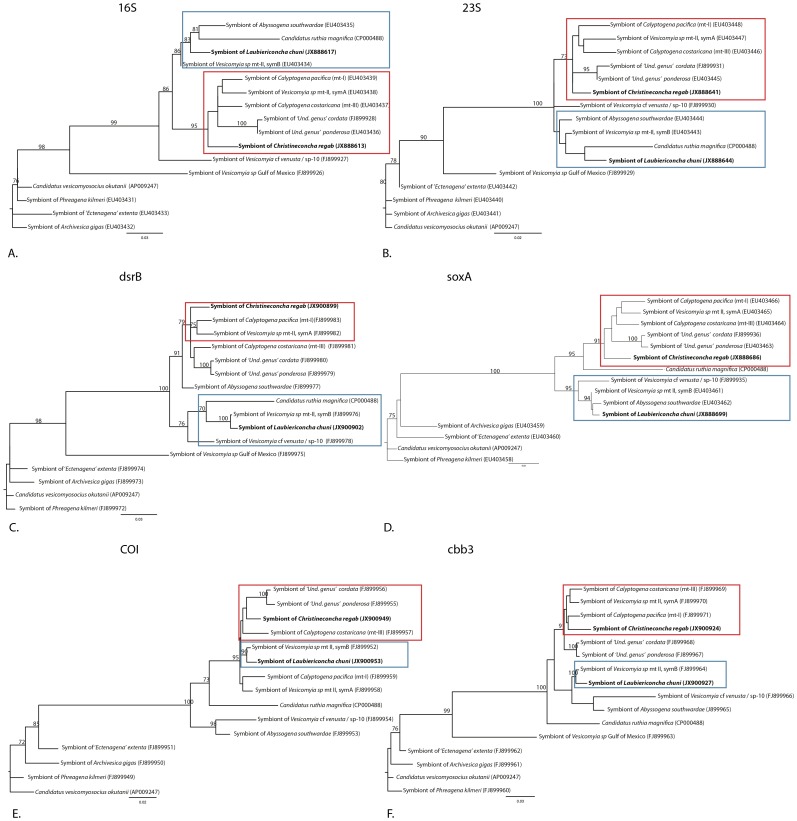
Unrooted maximum-likelihood phylogenies for six vesicomyid symbiont marker genes. Boxes indicate clade designations: *L. chuni*/*Vesicomya* sp. mtII symB (blue), *C. regab*/*Vesicomya* sp. mtI, mtII symA (red). Bootstrap values for 1000 replicates are given in percent above branches and clades (70% only). Scale bars are expressed as number of substitutions per base pair. The evolutionary model tested was GTR+I+G. with the following parameters: proportion of invariable sites = 0.79 (A), 0.56 (B), 0 (C, D, E, F), number of substitution rates (nst) = 6 for all phylogenies, gamma distribution parameter = 0.84 (A), 1.11 (B), 0.11 (C), 0.32 (D),0.15 (E), 0.14 (F). ‘Und. genus’) indicates a temporary genus name. Sp- 10 corresponds to the species numbers given in Audzijonyte *et al.* 2012.

All gene phylogenies obtained here, except that of *aprA*, displayed clear separation between sequences dominating in *C. regab* symbionts and those found in *L. chuni* symbionts (Figure 4). *C. regab* symbionts belonged to a clade including *Vesicomya* spp. mtI, mtII (SymA), *Calyptogena costaricana* (formerly *V.* sp. mtIII), and ‘Undetermined genus’ *cordata* and ‘Undetermined genus’ *ponderosa*, and the latter clustered with *Vesicomya* sp. mtII (SymB), *Abyssogena southwardae* (formerly *V.* sp. ‘Mid-Atlantic Ridge’) and *Vesicomya* cf *venusta* (formerly *V.* sp. BR) from Blake Ridge.

In the *aprA*-based phylogeny (Figure S2), both symbiont strains clustered with various vesicomyid-associated symbionts and with *Thyasira vulcolutre* symbionts, but groupings seen in other trees were not observed (Figure S2). However, as observed for other genes, the *C. regab* and *L. chuni* symbiont strains were still clearly different (4.9% difference in nucleic acid sequence).

### 4. Identification of symbionts and estimation of symbiont densities using FISH

**Figure 5:**
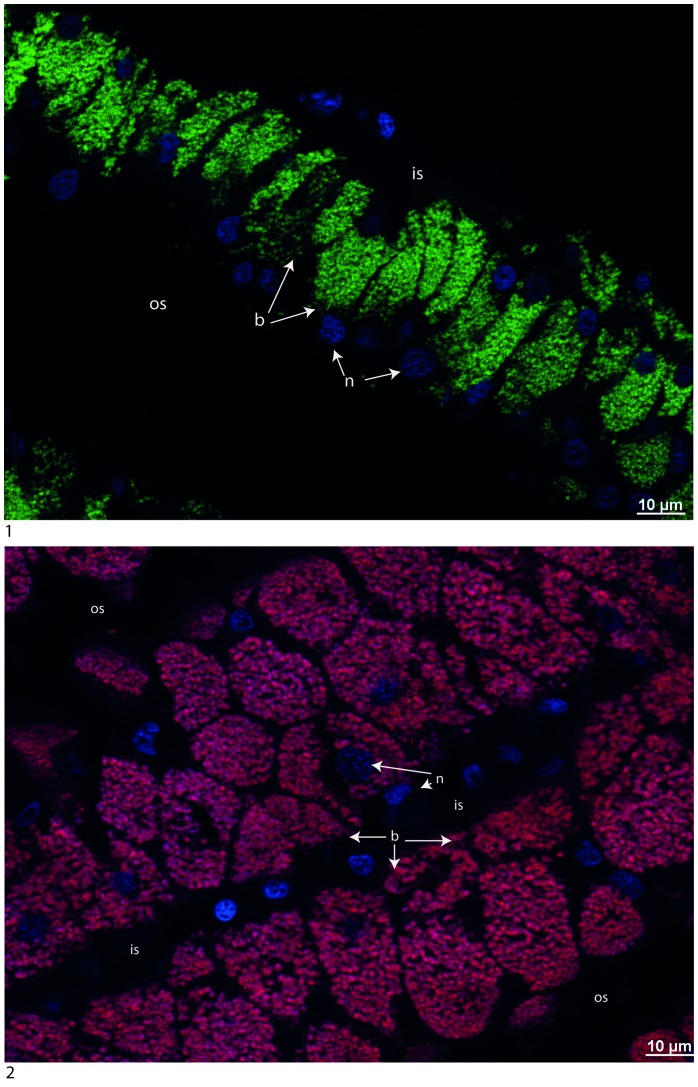
Cross-sections of gills dissected from *C. regab* (217-V4) (1) and *L. chuni* (225-V6) (2) and hybridized with Creg821 (Cy3, stained in green) and Lchun821 (Cy5, stained in red) FISH probes, and DAPI counterstained (in blue). For the individuals shown, only a single 16S rRNA symbiont phylotype was identified through PCR. Specific probes and DAPI (2) do not overlap perfectly, possibly due to low activity in some bacteria. b: bacteriocyte, n: nucleus, os: outer lamellar space, is: intralamellar space.

**Figure 6:**
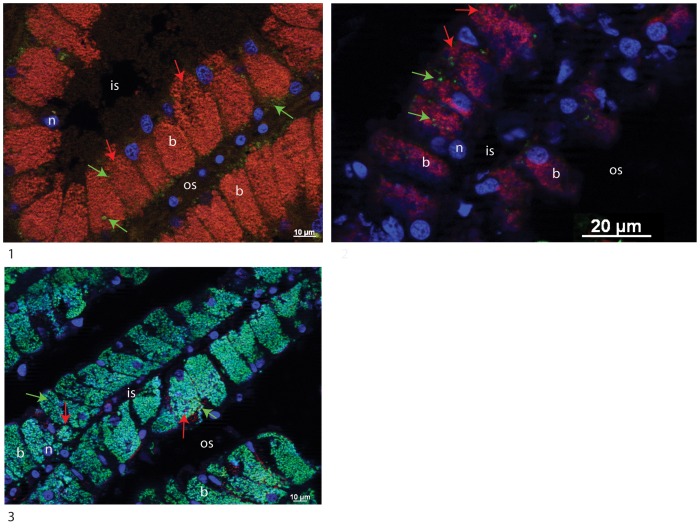
Cross-sections of gills dissected from *C. regab* (1 and 2) and *L. chuni* (3) (1:217-V2 and 2:225- V3 and 3:225-V1) and hybridized with Creg821 (Cy5, stained in red) and Lchun821 (Cy3, stained in green) FISH probes, and counterstained with DAPI (in blue). In addition to the expected symbiont 16S rRNA phylotype, the unexpected phylotype is visible. Arrows indicate bacteria hybridized with both Creg821 (red) and Lchu821 (green). For individuals shown, a single (1 and 2) or two symbionts (3) were detected with 16S rRNA PCR probes. Images from specific probes and DAPI staining(3) do not overlap perfectly, possibly due to low activity in some bacteria. b: bacteriocyte, n: nucleus, os: outer lamellar space, is: intralamellar space.

The presence of a single or both bacterial 16S rRNA phylotypes was investigated in the gill tissues of *C. regab* and *L. chuni*. Bacteria were abundant in the lateral zone of gill filaments in both host species and hybridized with the Eub338 probe. Probes Creg821 and Lchu821 successfully hybridized in symbionts of *C. regab* and *L. chuni*, respectively (Figure 5). For two specimens of *C. regab* from Sites 2 and 3, probe Lchu821 hybridized with low numbers of bacteria (Figure 6 and Figure S3). Similarly, one specimen of *L. chuni* also displayed low numbers of bacteria hybridized with probe Creg821 (Figure 6 and Figure S3). In these cases, the unexpected symbiont was rare compared to the expected symbiont, and signals from both probes did not overlap, confirming they targeted distinct bacterial strains. In Figures 5-2 and 6-3, DAPI and symbiont-specific probe signals were not perfectly overlaid, with certain bacteria displaying no signal from either probe. This may be due to low activity level of certain bacteria, or to the presence of additional bacterial strains not detected by either probe, and not detected in our 16S rRNA clone libraries. No Lchu821 signal was ever seen in any of the five *C. regab* specimens investigated from Site 1.

Bacteria occupied on average 64.3±17.2% of gill epithelial cell volume in *C. regab* and 51.3±12.4% in *L chuni*. Specimens of *L. chuni* displayed significantly lower densities of bacteria compared to *C. regab* (Wilcoxon-Mann Whitney, W = 68030, p0.0001). Density of bacteria also varied among sites for *C. regab*, with significantly higher density in individuals sampled from Site 2 (76.0±18.4%), than in Site 1 (53.3±11.9%) and Site 3 (63.6±12.4%) (Kruskal-Wallis test: X^2^ = 140.95, p0.0001).

## Discussion

### 1. Co-occurrence of two symbionts in Regab vesicomyids

Vesicomyid bivalves are known to harbor chemoautotrophic Gammaproteobacteria [35], [36] in their gills, within specialized cells, called bacteriocytes [6], [37], [38]. In accordance, BLAST searches revealed that the two 16S rRNA phylotypes identified in the present study belonged to this bacterial group. Each bacterial phylotype was characteristic of one host vesicomyid species, with co-occurrence of both phylotypes in some specimens of each host species.

At the Regab pockmark, a previous study identified a single 16S rRNA phylotype of sulfide-oxidizing bacteria in one of the two vesicomyid species sampled there [39]. Here, our study revealed two 16S rRNA phylotypes from the 27 specimens of vesicomyids investigated. One phylotype was present in all specimens of *C. regab*, and the second was present in all specimens of *L. chuni*. Results of *in situ* hybridizations using phylotype-specific probes confirmed that these two phylotypes dominated the symbiont population in gill epithelial cells. Neither of the two phylotypes corresponded to the one previously observed at Regab [39], which appeared to be a chimera of the two phylotypes identified in this study. This is a common pitfall of the PCR technique when amplifying similar sequences. Because no background knowledge was available in 2009 regarding symbiont diversity, this sequence had not been initially diagnosed as a chimera.

All the other bacterial genes investigated (23S rRNA, *aprA*, *dsrB*, *soxA*, *ccb3*, *coxI*) also showed two different sequences: one present in all *C. regab* and another in all *L. chuni* specimens, and the most parsimonious deduction is that they belong to the same two bacterial strains characterized by their 16S rRNA phylotypes. However, both phylotypes were shown to co-occur in several vesicomyid specimens for one (*C. regab* 217-V2 and *L. chuni* 225-V6) or two genes (*C. regab* 217-V3, 225-V5 and *L. chuni* 225-V1), although the sequence characteristic of the host species always dominated. Interestingly, some specimens showing both 16S rRNA phylotypes showed a single expected sequence at all other marker genes, whereas some specimens for which no admixture of 16S rRNA phylotypes had been detected harbored both sequences of one or two other marker genes in the MLST analysis (Table 3). Several hypotheses can be proposed to explain such patterns of co-occurrence despite the consistent dominance of the expected bacterial strain. Hybridization between the two distinct host species may have favored co-acquisition of paternal and maternal bacterial lineages, as observed for mitochondria in some bivalves, but this is not the most parsimonious hypothesis. Indeed, these two vesicomyid species are phylogenetically very divergent (Figure S1 and [18]), and it is thus very unlikely that they can hybridize. Moreover, paternal symbiont or mitochondrial transfer has not been reported in vesicomyids [16].

A more likely hypothesis would be that the two symbionts actually co-occur in some specimens as a result of occasional lateral acquisition of another symbiont strain in addition to the maternally transmitted symbiont strain. Alternatively, erratic detection of distinct lineages only at some loci may seemingly reveal the occurrence of mosaic symbiont strains derived from ancient events of partial recombination between distinct symbiont strains.

Three lines of evidences favor the symbiont co-occurrence hypothesis over the occurrence of anciently recombined symbionts. First, when an unexpected strain is identified in a single specimen for a particular gene (*e.g.* 217-V3), the expected lineage is also detected for this gene and the other genes, with only one exception (*coxI* in 225-V3). However, each of the genes sequenced here are, to the best of our knowledge, single-copy genes in vesicomyid symbiont genomes, so the occurrence of two different copies of the gene suggests symbiont co-occurrence, not recombination [17], [40], [41]. Second, recombination is unlikely between symbionts because gene-based phylogenetic trees show that the two sequences of each gene belong to two separate clades. Any recent recombination between these groups would have resulted in a different branching order, with unexpectedly closer similarity between the sequences of a given gene compared to that of other genes, which was not observed here (Figure 4). Finally, FISH observations confirmed the low abundance of the unexpected symbiont in at least three specimens (two *C. regab* and one *L. chuni*) (Figure 6). In these cases, probe signals did not overlap, confirming that each bacterium expressed only a single 16S rRNA sequence. In several FISH images, some signals from minority symbionts appeared to be located in the most basal part of the bacteriocytes, and surrounded by the dominant, expected symbiont. This suggests intracellular localization, although this issue cannot be settled without further TEM investigations involving correlative light and FISH-based symbiont identification along with ultrastructural studies. As these unexpected symbionts were very rare compared to the ones characteristic of each species, it is likely that they were present in more specimens than detected here. For example, none of the co-occurrences inferred from PCR results were confirmed by FISH observations on the same specimens. This is possibly due to the rarity of unexpected symbionts combined with a relatively low sampling effort for the FISH analysis, because only a few sections per specimen were observed. FISH observations revealed the co-occurrence of two symbiont 16S rRNA phylotypes in specimens in which PCR tests failed to detect the unexpected phylotype (*e.g.* 217-V2, Figure 5, Table 3). Co-occurring symbionts may thus be erratically detected (or not) through direct PCR and PCR-cloning-based approaches due to the preferential amplification of the dominant phylotype, and may be erratically detected in FISH-based tests because of the low abundance of unexpected symbionts. Larger clone libraries, and investigation of many more FISH slides would certainly increase the probability of detecting the second 16S rRNA phylotype in specimens. In any case, the unexpected phylotype was always rare compared to the expected one.

Previously considered as exclusively maternally transmitted [14], [15], [42], [43], vesicomyid symbionts may also be horizontally transferred from another host [16], resulting in two co-occurring symbiont strains within a single host specimen [44]. Here, MLST approaches and FISH observations both confirmed the dominance of the expected symbiont in all specimens, but also strongly suggest that the second symbiont can be present in low amounts in the gills, whereas genetic recombination among symbionts is not supported by our data.

### 2. Co-occurrence of Vesicomyidae hosts seems to favor lateral acquisition of symbionts

Interestingly, no evidence was found for co-occurrence in *C. regab* specimens from Site 1, with either PCR or FISH. Among the six specimens displaying phylotypes or FISH observations from both symbionts, four were sampled from Site 3 where *C. regab* and *L. chuni* co-occurred in the same clam bed. The two other specimens were from Site 2 where only *C. regab* was collected during the 2008 Guineco cruise, but *L. chuni* was observed three years later during another cruise (WACS, 2011), indicating that both species actually also co-occur at this site. All *C. regab* from Site 1, in which no *L. chuni* has ever been sampled, only displayed the typical *C. regab*-associated bacterial sequences and FISH signals. Mosaics of marker gene sequences were only detected at sites where both vesicomyid species are known to co-occur, suggesting that host co-occurrence favors symbiont co-occurrence. This fits with the observation that vesicomyid symbionts are predominantly maternally inherited, and genomic data suggest a limited potential for free-living in these bacteria. The two sequenced genomes available from symbionts of *Phreagena okutanii* and ‘Undetermined genus’ *magnifica* have considerably reduced genome sizes, of only 1.02 and 1.2 Mb, respectively [40], [45], [46]. Several important genes for DNA recombination and repair have been lost, possibly as an adaptation to the intracellular environment [40], [46], [47]. Similarly, several important genes involved in motility are absent. Vesicomyid symbionts form a tight clade in 16S rRNA-based phylogenies in which there are no free living environmental strains, further supporting they are at least very rare in the environment. If no free-living form occurs, physical proximity (or co-occurrence within a single bed) of Vesicomyidae hosts would be the only way distinct symbiont strains could encounter each other.

The evolutionary consequences of occasional lateral acquisition of symbionts are multiple. For bacteria, such occasional ‘leakage’ could favor exchange of genetic material between symbionts, for which other studies have provided evidence [17], although we consider such event of recombination as unlikely in the case reported here due to deep divergence among dominant symbionts strains. Bringing together both symbionts within a single host could also result in inter-symbiont competition, potentially leading to symbiont displacement. Symbiont displacement can occur if the symbiont transmitted to offspring is not the one that was initially inherited maternally. This requires that a laterally acquired symbiont becomes more efficiently transmitted to the next generation than the maternally inherited symbiont, resulting in a replacement event analogous to genomic “selective sweeps” [48] on this non-genomic but heritable compartment. To test this, presence and abundance of non-specific symbionts need to be investigated in the reproductive tissues, gametes and larvae of Vesicomyidae, and their likelihood of being transmitted needs to be estimated. No evidence for recombination or lineage sorting due to competition between dominant and minority symbionts was observed in our dataset, suggesting that incidental leakage has little consequence. However, even if rare, events of symbiont displacement may have occurred over longer evolutionary times. They could be detected as incongruencies between host and symbiont phylogenies, emphasizing the importance of including as many species as available in future host-symbiont phylogenetic studies.

Overall, physical proximity (or co-occurrence in a single bed) of Vesicomyidae hosts may be a driving force for the evolution of their symbionts either toward displacement or emergence of new mosaic lineages through recombination. For hosts, this opens the possibility of acquiring locally-adapted symbiont strains already present in another species thriving in a habitat. Because genetic exchanges can occur either within hosts or in the environment, the existence of free-living or dormant forms of vesicomyid symbionts in the environment should be further investigated.

### 3. Symbiont densities and characteristics of the habitats

At the Regab pockmark, *C. regab* and *L. chuni* each harbored a distinct, dominant symbiont. Symbiont quantification using 3D-FISH indicated that *C. regab* specimens from the three sampling sites have distinct symbiont densities. At Site 2, methane and sulfide fluxes and concentrations were higher than at the Site 3, both in 2001 and 2008 [12], [49], [50]. Interestingly, symbiont densities were higher at the Site 2 (76%) than at the two other sites (63.6% at the south-west site and 53.3% at the north site), suggesting a possible relationship between symbiont densities and local sulfide availability within a given species, and thus plasticity in the symbiotic interaction depending on environmental conditions. Such intraspecific variability in symbiont densities in relation to the chemical characteristics of the habitat has been documented in dual-symbiotic mytilids of the genus *Bathymodiolus*, including at the Regab site [19], [51]. This variability appears to contribute to optimizing resource use (carbon uptake) with respect to the cost of symbiosis (oxygen use, sulfide toxicity). In the present case, despite the fact that only sulfur-oxidizing symbionts were present in *C. regab*, their densities in animal tissue may be linked to sulfide availability.

*C. regab* and *L. chuni* co-occur in the same sampled bed at Site 3, but there *L. chuni* specimens displayed lower symbiont densities (51.2 *vs.* 64.3%) than *C. regab*. The two vesicomyids have slightly different micro-habitats. *C. regab* is buried in the sediment with the anterior half or two-thirds of the shell visible on the surface, whereas *L. chuni* is totally buried with only the tip of its very long siphons visible on the surface [9]. With its foot extended, *L. chuni* can reach sulfide sources deeper in sediment and more sulfide is available deeper at Site 3 compared to other sites [50]. This slightly different micro-habitat possibly results in a more limited access to oxygen for hosts and symbionts. Although it is a less efficient electron acceptor, nitrate can be used as an energy source in symbionts of *Lucinoma aequizonata* [52] and the solemyid *Solemya reidi* [53]. In vesicomyids, some symbiont genomes possess the genes necessary for respiration on nitrate, while others do not. Symbionts of *Phreagena okutanii* and *P. kilmeri* have a functional dissimilatory nitrate reductase that is absent in symbionts of *‘Undetermined genus’ magnifica*, for example [54]. These results suggest differences in host geochemical ecology [41], [54]–[56], which may contribute to niche separation. In our case, niche separation may explain both the lower overall densities of symbionts (less energy gained using nitrate as an acceptor), and the lower abundance of *L. chuni* compared to *C. regab*. In this respect, the red hemolymph of *L. chuni*, suggesting the presence of hemoglobin, which is absent in *C. regab*, may compensate for part of the handicap resulting from its hypoxic habitat ([57], pers. obs. of authors). Differences in symbiont densities have been demonstrated between two species living in the Monterey Bay and are linked, among other things, to micro-habitat chemistry variability. *C. pacifica* inhabits areas of lower environmental sulfide levels whereas *P. kilmeri* dominates in areas with higher sulfide levels [13]. However, the density of bacteria per gram body weight in *C. pacifica* was higher than in *P. kilmeri* [13]. Nevertheless, *L. chuni* possesses double gills (two demibranchs) and they generally weigh 40% more than *C. regab* (unpublished data). Therefore, the double gills may enhance sulfide fixation capability compared to *C. regab*, despite the lower symbiont densities per unit gill volume. At this stage, it is difficult to conclude, and more quantitative estimates of *in situ* sulfide and oxygen concentrations are required to interpret correctly the differences reported herein between *C. regab* and *L. chuni*. Nevertheless, the depth segregation in the sediment column, together with differences in symbiont strains and densities, as well as their presence in distinct chemical conditions, probably indicate that the two species have slightly different ecological niches, which probably limits interspecific competition, and could lead to co-occurrence of the species within a single bed under certain conditions.

## Conclusion

*C. regab* and *L. chuni* from the Regab pockmark each have one species-specific, dominant symbiont strain. However, host species can harbor the symbiont from the other species in low amounts when hosts co-occur within the same vesicomyid bed. Sympatry may therefore be important for favoring exchange or displacement of otherwise maternally transmitted symbiont strains, or to promote genetic exchanges between symbionts over evolutionary time. Whatever the cause, symbiont co-occurrence reveals that vertical transmission is “leaky” in some aspects. The co-occurrence of host species in single beds probably favors symbiont exchanges which in turn may contribute to homogenizing the symbiont populations over time, thereby explaining the high level of similarity among symbionts from various vesicomyid hosts compared to the ancient radiation of the Vesicomyidae family [58] (16S rRNA sequence similarity is often greater than 96% despite the fact that that the group is 50 to 80 million years old). Symbiont densities were different between different clam beds for a given species, suggesting the influence of local environmental factors such as the availability of sulfide or oxygen for example. Differences in densities were also seen in co-occurring species within a given patch, possibly linked with differences in host habitat (surface vs. deeper sediment), anatomy (single vs. double demibranchs) or physiology (presence of hemoglobin). This slight niche differentiation promotes species co-occurrence. Occasional acquisition of non-parental symbiont strains favored by host species co-occurrence may promote recombination and symbiont displacement over time in Vesicomyidae. To test this, future studies should evaluate the vertical transmission of laterally-acquired bacteria, and estimate rates of symbiont recombination and displacement using phylogenetic approaches on a the broadest panel of species available.

## Supporting Information

Figure S1 DNA maximum-likelihood tree on vesicomyid hosts based on *coxI* nucleotide sequences rooted with *Venus antiqua*, due to its close relationship with vesicomyids. The evolutionary model tested was GTR+I+G [30] (proportion of invariant sites = 0.48, number of substitution rates = 6, gamma distribution parameter = 0.56). Bootstrap values for 1000 replicates are given in percent above branches and clades. Specimens are named according to recent species revisions. Scale bars are expressed as the number of substitutions per base pair. ‘Und. genus’) indicates a temporary genus name. Sp-1 to 10 correspond to species numbers given in Audzijonyte *et al.* 2012.
(TIF)

Figure S2 *aprA* maximum-likelihood tree for symbiont bacteria. The evolutionary model tested was GTR+I+G (proportion of invariable sites = 0.22, number of substitution rates = 6, gamma distribution parameter = 0.72). Bootstrap values for 1000 replicates are given in percent above branches and clades (70% only). Scale bars expressed as the number of substitutions per base pair.
(TIF)

Figure S3 Cross-sections of gills dissected from *C. regab* (1) and *L. chuni* (2) (1: 225-V3 and 2: 225-V1) with individual channels for DAPI (in blue) (a), Creg821 (stained in red) (b), Lchun821 (stained in green) (c) and a composite image of all three channels (d).
(TIF)

Table S1 Primers used for gene amplifications.
(DOC)

Table S2 Genbank accession number.
(DOC)
