# Supplementary figures and images for: Physical Proximity May Promote Lateral Acquisition of Bacterial Symbionts in Vesicomyid Clams
Source: PLoS One. 2013 Jul 8;8(7):e64830. doi: 10.1371/journal.pone.0064830 (PMC3704533; doi:10.1371/journal.pone.0064830)

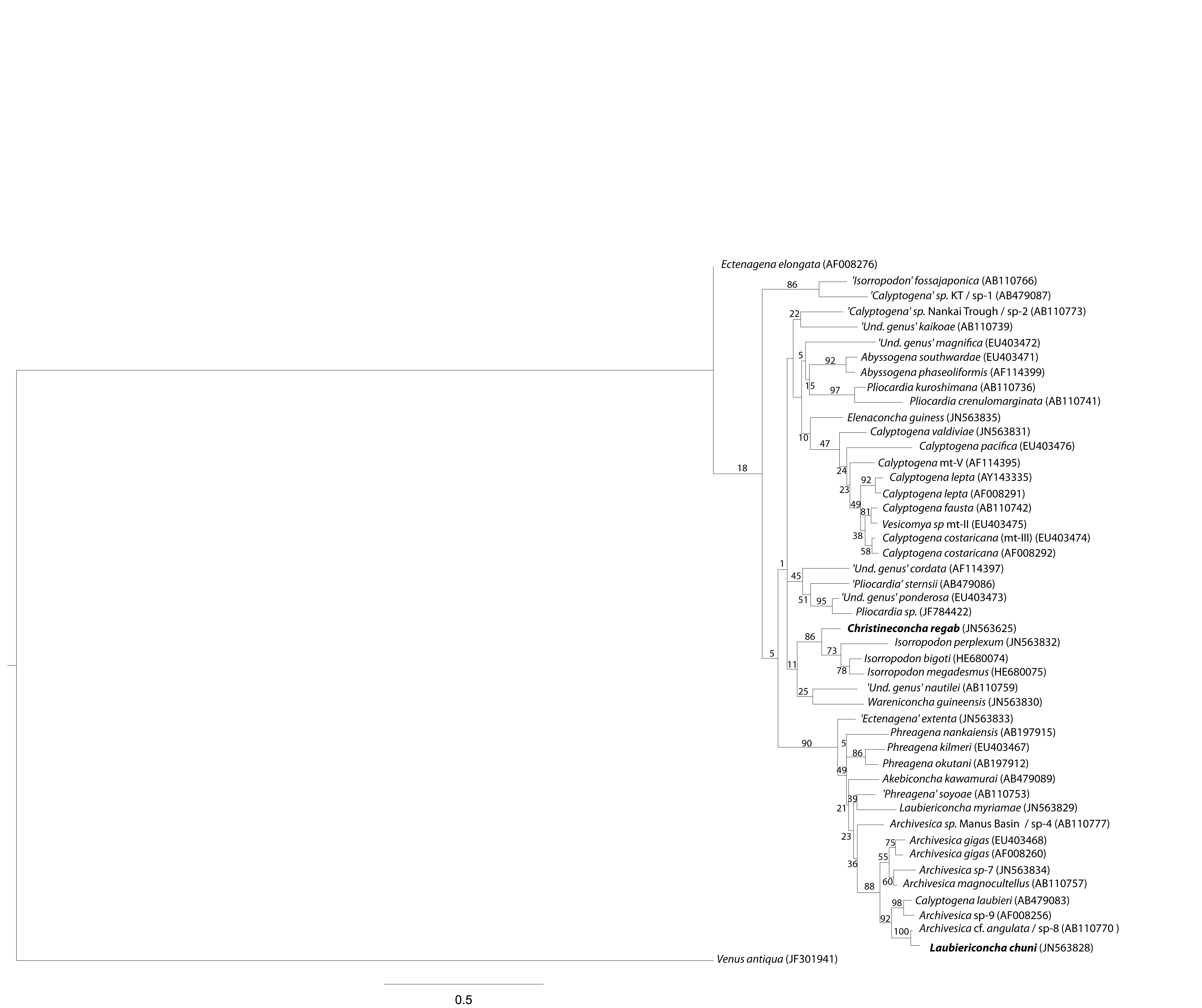

Supplement: Figure S1 — DNA maximum-likelihood tree on vesicomyid hosts based on coxI nucleotide sequences rooted with Venus antiqua, due to its close relationship with vesicomyids. The evolutionary model tested was GTR+I+G [30] (proportion of invariant sites = 0.48, number of substitution rates = 6, gamma distribution parameter = 0.56). Bootstrap values for 1000 replicates are given in percent above branches and clades. Specimens are named according to recent species revisions. Scale bars are expressed as the number of substitutions per base pair. ‘Und. genus’) indicates a temporary genus name. Sp-1 to 10 correspond to species numbers given in Audzijonyte et al. 2012. (TIF) [file pone.0064830.s001.tif]

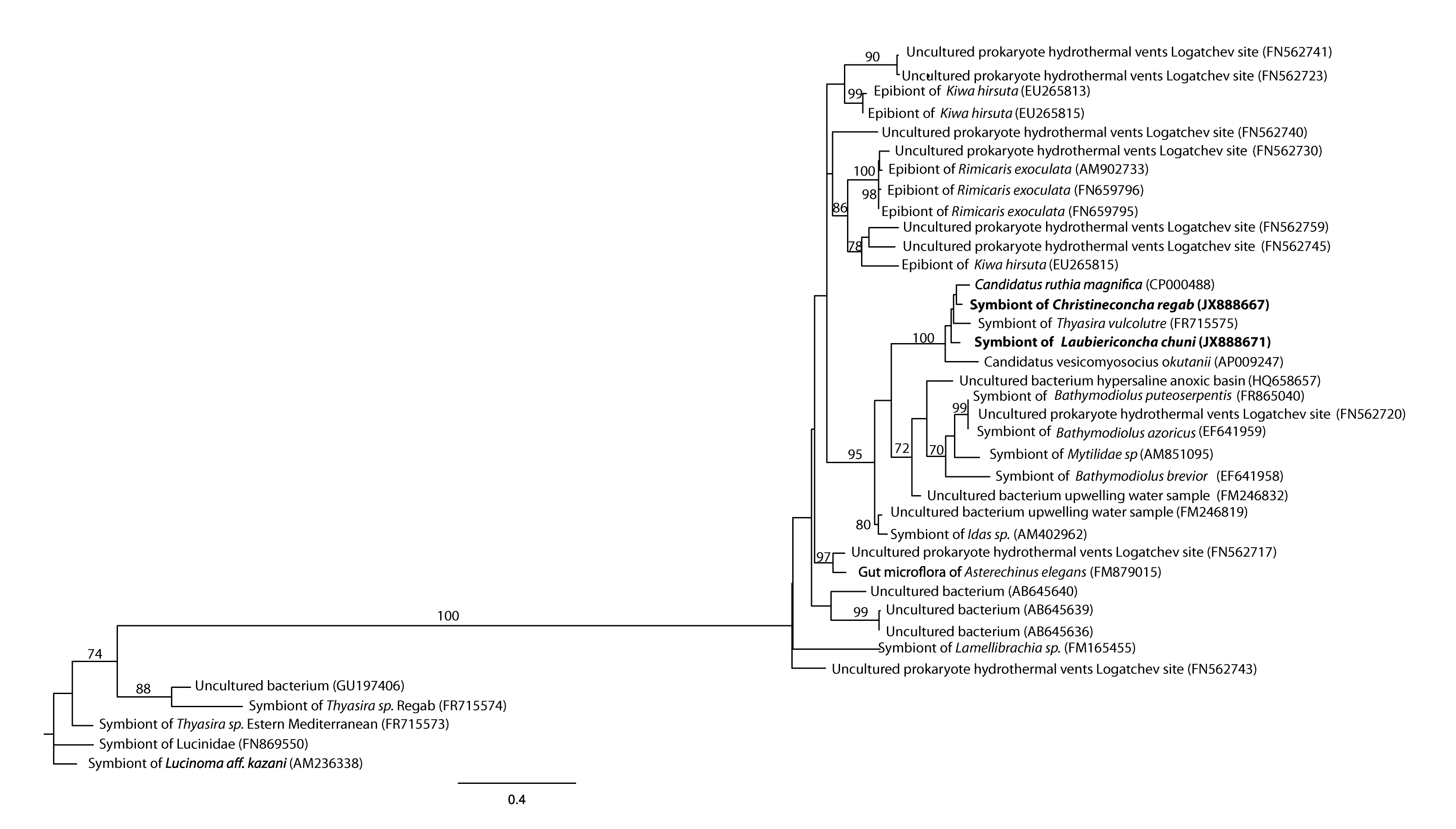

Supplement: Figure S2 — aprA maximum-likelihood tree for symbiont bacteria. The evolutionary model tested was GTR+I+G (proportion of invariable sites = 0.22, number of substitution rates = 6, gamma distribution parameter = 0.72). Bootstrap values for 1000 replicates are given in percent above branches and clades (70% only). Scale bars expressed as the number of substitutions per base pair. (TIF) [file pone.0064830.s002.tif]

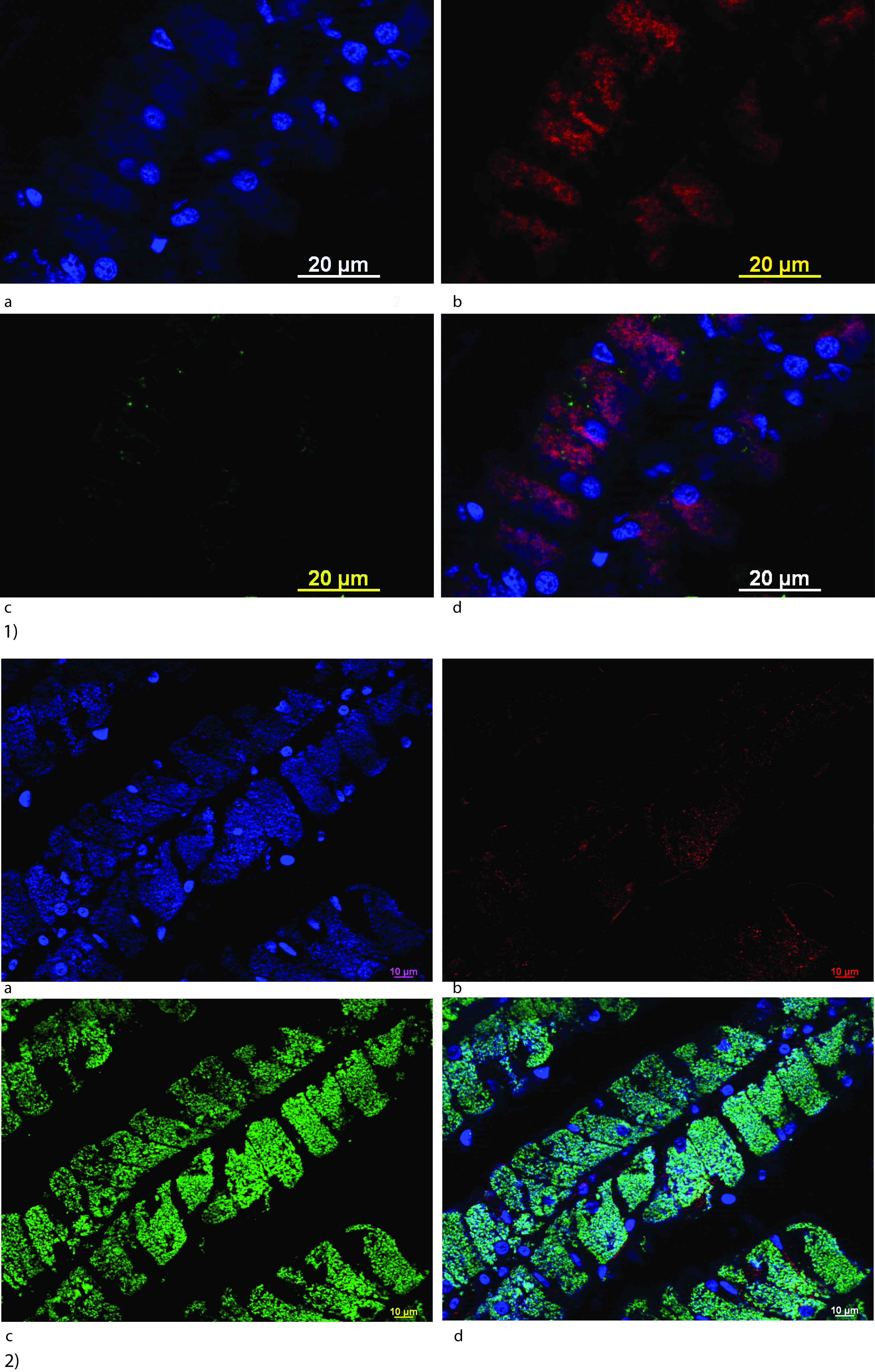

Supplement: Figure S3 — Cross-sections of gills dissected from C. regab (1) and L. chuni (2) (1: 225-V3 and 2: 225-V1) with individual channels for DAPI (in blue) (a), Creg821 (stained in red) (b), Lchun821 (stained in green) (c) and a composite image of all three channels (d). (TIF) [file pone.0064830.s003.tif]
