# Supplementary material for: Physical Proximity May Promote Lateral Acquisition of Bacterial Symbionts in Vesicomyid Clams
Source: PLoS One. 2013 Jul 8;8(7):e64830. doi: 10.1371/journal.pone.0064830 (PMC3704533; doi:10.1371/journal.pone.0064830)
Supplement: Table S1 — Primers used for gene amplifications. (DOC) [file pone.0064830.s004.doc]

**Table S1**: Primers used for gene amplifications.

| Locus | Gene/Region | Function | | | Primers (5’-3) | | Annealing (cycles) | | References | |
| --- | --- | --- | --- | --- | --- | --- | --- | --- | --- | --- |
| 16S | Small SubunitrRNAgene | Ribosomal RNA | | 27-F: AGAGTTTGATCATGGCTCAG  1492-R: ACGGCTACCTTGTTACGACTT | | 45°C (27) | | Muyzer et al. 1995 | |  |
| 23S | Large SubunitrRNAgene | Ribosomal RNA | | 3505-F: GACCGTCAGCTAAGGTCCCAA  4181-R : CTAGTTCCTTCACCCGAGTTCTC | | 53°C (35) | | Stewart et al. 2009 | |  |
| APS | sulfideoxidation | Sulfurmetabolism | | APS1-F: TGGCAGATCATGATYMAYGG  APS4-R: GCGCCAACYGGRCCRTA | | 58°C (25) | | Mayer and Kuever 2007 | |  |
| soxA | Sulfuroxidationprotein | Sulfurmetabolism | | soxA-F: CCACAGTGGCTAGTTTGGGTTTGT  soxA-R: TCAAACAACCATCATAACGACGATG | | 54°C (35) | | Stewart et al. 2009 | |  |
| dsrB | Sulfite reductase, dissimilatory-type beta subunit | Sulfurmetabolism | | dsrB-F: GGCTGAAGCACCAAGAATGCCTATC  dsrB-R: GCTGGCCAACCAATACGATC | | 54°C (25) | | Stewart et al. 2009 | |  |
| COI | Cytochrome c oxidase subunit I | Energy transduction | | symCOI-F: CCATCACGGTCCTGAAAAAGGC  symCOI-R: GCGTACATAAAATACAACTCACC | | 51°C (25) | | Stewart et al. 2009 | |  |
| cbb3 | cbb3-type cytochrome c oxidase subunit I | | Energy transduction | cbb3-F: GGTGTTGTTAAATGGTTTGC  cbb3-R : CATGACCAATCGTCCAATC | | 48°C (25) | | Stewart et al. 2009 | |  |
