# Supplementary material for: Physical Proximity May Promote Lateral Acquisition of Bacterial Symbionts in Vesicomyid Clams
Source: PLoS One. 2013 Jul 8;8(7):e64830. doi: 10.1371/journal.pone.0064830 (PMC3704533; doi:10.1371/journal.pone.0064830)
Supplement: Table S2 — Genbank accession number. (DOC) [file pone.0064830.s005.doc]

**Table S2**: Genbank accession number.

| Gene |  | Host | Symbiont | | | | | | |
| --- | --- | --- | --- | --- | --- | --- | --- | --- | --- |
|  |  | COI | 16S | 23S | APS | soxA | dsrB | cbb3 | COI |
| Specimen | Species |  |  |  |  |  |  |  |  |
| 209-V1 | *C. regab* | jx900960 | jx888597 | jx888625 | jx888651 | jx888680 | jx900883 | jx900909 | jx900934 |
| 209-V2 | *C. regab* | jx900961 | jx888598 | jx888626 | jx888652 | jx888681 | jx900884 | jx900910 | jx900935 |
| 209-V3 | *C. regab* | jx900962 | jx888599 | jx888627 | jx888653 | jx888682 | jx900885 | jx900911 | jx900936 |
| 209-V4 | *C. regab* | jx900963 | jx888600 | jx888628 | jx888654 | jx888683 | jx900886 | jx900912 | jx900937 |
| 209-V5 | *C. regab* | jx900964 | jx888601 | jx888629 | jx888655 | jx888684 | jx900887 | jx900913 | jx900938 |
| 211-Net5-V1 | *C. regab* | JN563825 | jx888602 | jx888630 | jx888656 | jx888689 | jx900888 | jx900914 | jx900939 |
| 211-Net5-V2 | *C. regab* | jx900965 | jx888603 | jx888631 | jx888657 | jx888690 | jx900889 | jx900915 | jx900940 |
| 211-Net5-V3 | *C. regab* | jx900966 | jx888604 | jx888632 | jx888658 | jx888691 | jx900890 | jx900916 | jx900941 |
| 211-Net5-V4 | *C. regab* | jx900967 | jx888605 | jx888633 | jx888659 | jx888692 | jx900891 | jx900917 | jx900942 |
| 211-Net5-V5 | *C. regab* | jx900968 | jx888606 | jx888634 | jx888660 | jx888693 | jx900892 | jx900918 | jx900943 |
| 211-PC12-V1 | *C. regab* | jx900969 | jx888607 | jx888635 | jx888661 | jx888694 | jx900893 | jx900919 | jx900944 |
| 211-PC12-V2 | *C. regab* | jx900970 | jx888608 | jx888636 | jx888662 | jx888695 | jx900894 |  | jx900945 |
| 211-PC12-V3 | *C. regab* | jx900971 | jx888609 | jx888637 | jx888663 | jx888696 | jx900895 | jx900920 | jx900946 |
| 211-PC12-V4 | *C. regab* | jx900972 | jx888610 | jx888638 | jx888664 | jx888697 | jx900896 | jx900921 | jx900947 |
| 211-PC12-V5 | *C. regab* | JN563826 | jx888611 | jx888639 | jx888665 | jx888698 | jx900897 | jx900922 | jx900948 |
| 217-V2 | *C. regab* | jx900959 | jx888612 | jx888640 | jx888666 | jx888685 | jx900898 | jx900923 | jx900949 and JX900950 |
| 217-V3 | *C. regab* | jx900973 | jx888613 and jx888614 | jx888641 | JX888667 and JX888668 | jx888686 | jx900899 | jx900924 | ND |
| 217-V4 | *C. regab* | JN563827 | jx888615 | jx888642 | jx888669 | jx888687 | jx900900 | jx900925 | jx900951 |
| 217-V5 | *C. regab* | jx900974 | jx888616 | jx888643 | jx888670 | jx888688 | jx900901 | jx900926 | jx900952 |
| 225-V1 | *L. chuni* | JN563828 | jx888617 and jx888618 | jx888644 | JX888671 and JX888672 | jx888699 | jx900902 | jx900927 | jx900953 |
| 225-V2 | *C. regab* | jx900975 | jx888619 | jx888645 | jx888673 | jx888700 | jx900903 | jx900928 | ND |
| 225-V3 | *C. regab* | jx900976 | jx888620 | jx888646 | jx888674 | jx888701 | jx900904 | jx900929 | jx900954 |
| 225-V4 | *C. regab* | jx900977 | jx888621 | jx888647 | jx888675 | jx888702 and JX888703 | jx900905 | jx900930 | jx900955 |
| 225-V5 | *C. regab* | jx900978 | jx888622 | jx888648 | JX888676 and JX888677 | jx888704 | jx900906 | jx900931 | ND |
| 225-V6 | *L. chuni* | jx900979 | jx888623 | jx888649 | jx888678 | jx888705 | jx900907 | jx900932 | JX900956 and jx900957 |
| 225-V7 | *L. chuni* | jx900980 | jx888624 | jx888650 | jx888679 | jx888706 | jx900908 | jx900933 | jx900958 |
